# Acquisition of Motor and Cognitive Skills through Repetition in Typically Developing Children

**DOI:** 10.1371/journal.pone.0158684

**Published:** 2016-07-06

**Authors:** Sara Magallón, Juan Narbona, Nerea Crespo-Eguílaz

**Affiliations:** 1 School of Psychology, Faculty of Medicine and Health, University of Leeds, Lifton Place, LS2 9JZ, Leeds, West Yorkshire, United Kingdom; 2 Faculty of Education and Psychology, University of Navarra, Campus Universitario, 31080, Pamplona, Navarra, Spain; 3 Unit of Paediatrics Neurology, Faculty of Medicine, University of Navarra, Av. de Pío XII, 36, 31008 Pamplona, Navarra, Spain; Waseda University, JAPAN

## Abstract

**Background:**

Procedural memory allows acquisition, consolidation and use of motor skills and cognitive routines. Automation of procedures is achieved through repeated practice. In children, improvement in procedural skills is a consequence of natural neurobiological development and experience.

**Methods:**

The aim of the present research was to make a preliminary evaluation and description of repetition-based improvement of procedures in typically developing children (TDC). Ninety TDC children aged 6–12 years were asked to perform two procedural learning tasks. In an assembly learning task, which requires predominantly motor skills, we measured the number of assembled pieces in 60 seconds. In a mirror drawing learning task, which requires more cognitive functions, we measured time spent and efficiency. Participants were tested four times for each task: three trials were consecutive and the fourth trial was performed after a 10-minute nonverbal interference task. The influence of repeated practice on performance was evaluated by means of the analysis of variance with repeated measures and the paired-sample test. Correlation coefficients and simple linear regression test were used to examine the relationship between age and performance.

**Results:**

TDC achieved higher scores in both tasks through repetition. Older children fitted more pieces than younger ones in assembling learning and they were faster and more efficient at the mirror drawing learning task.

**Conclusions:**

These findings indicate that three consecutive trials at a procedural task increased speed and efficiency, and that age affected basal performance in motor-cognitive procedures.

## Background

From the simplest act of clapping to the complexity of playing the violin, all require the ordering and timing of actions [1]. “The acquisition of a new motor skills normally relies on several phases of learning, including a fast early learning stage, a slow later stage, consolidation, automaticity and retention” [2, 3]. Procedural memory and learning allows acquisition and consolidation of motor skills and contributes to the use of those skills [4–6] and to the establishment of cognitive routines [6, 7]. In contrast to declarative memory, which refers to memories which can be consciously recalled, such as, facts and knowledge, and is concerned with what something is, procedural memory is concerned with behavioral and cognitive action, i. e how to do things [8]. Procedural memory does not require intentionality for retrieval [9]; given the appropriate cue, the procedural memory pops into mind whether we intend or not, even when we may be busy with other activities requiring attention [10].

That declarative and procedural memory skills are fundamentally separate is indicated by the occurrence of dissociation between them: it is widely found and reported that the procedural memory skills of patients suffering from anterograde amnesia are still intact [11–15] whereas declarative memory skills are impaired. In contrast, children with motor and coordination difficulties often retain declarative memory [16–19].

The fact that the performance at a given activity improves with repetition indicates that learning is involved in procedural tasks [20, 21]. If this were not the case, performance in ordinary everyday actions would consume disproportionate amounts of cognitive resources (i.e. actualized consciousness, or attention), preventing simultaneous involvement in other tasks. Similarly, if procedures were not retained in memory, they would take much longer to implement.

Automation of a cognitive or motor procedure is achieved through repeated practice. The performance of an expert at a procedure is characterized by mastery of the skill and a consequent release of cognitive resources for other tasks [22]. Therefore, for a given task, in addition to the effectiveness and efficiency of strategies, what differentiates an experienced practitioner from a novice is that the former can do other actions at the same time.

In performing a learned procedural task, fluent movement is conducted using open-loop (feed-forward) control and is attained in an automated manner. In learning a new procedural task, more reliance on feedback information is required. The use of a closed-loop feedback control requires practice to attain automation of movement. The notion of open- and closed-loop controls has been applied to the study of motor control in fine motor learning skills as handwriting [23–25].

In children, experience combined with neurobiological development results in the improvement of procedural skills (as well as other skills) [26, 27]. Children learn some procedures naturally through repetition or trial and error, with little cognitive effort. Other procedures can be acquired whilst playing games, or at school as part of a more systematic training based on repetition. Children are often unaware of the progressive development of their procedural skills and typically cannot specify exactly what they are doing or list the steps they follow. That is, children demonstrate that they can do something (i.e. their procedural learning) but may also demonstrate a lack of knowledge about how they do it (i.e. a lack of explicit learning) [28, 29].

## Methods

### Participants

Ninety typically developing Spanish children aged between 6 and 12 years who were randomly recruited from schools in Pamplona and Zaragoza (Spain) were included in the present study. Sex and age data are shown in Table 1. All participants came from families with a Hollingshead socioeconomic index [30] between two and three. Children schooled in a class below that corresponding to their age and children with an intelligence quotient below 85, as measured by the Peabody Picture Vocabulary Test-PPVT-III [31], were excluded. The PPVT-III was used in this case because it demands less time to be managed than other intelligence scales and it has been found to be strongly correlated with both verbal and nonverbal Intelligence Quotient (IQ) as measured by the Wechsler Intelligence Scale for Children -WISC-III- [32], the Kaufman Adolescent and Adult Intelligence Test -KAIT- [33] and the Kaufman Brief Intelligence Test -K-BIT- [34]. However, it would have been interesting to measure the intelligence by a more comprehensive test, including nonverbal skills.

**Table 1:** Description of the sample by sex and age.

Age (years)	Male		Female		Sex ratio (male/female)
	*n*	*%*	*n*	*%*	
6 (n = 17)	8	47	9	53	0.9
7 (n = 11)	6	55	5	45	1.2
8 (n = 13)	7	54	6	46	1.2
9 (n = 16)	7	44	9	56	0.8
10 (n = 12)	8	67	4	33	2
11 (n = 11)	8	73	3	27	2.7
12 (n = 10)	5	50	5	50	1
6–12 (N = 90)	49	54	41	46	1.2

All children participated voluntarily and informed written consent was obtained from their parents. The ethics committee of the Clínica Universidad de Navarra gave prior approval of the study.

### Measures

Two standard procedural learning tasks were adapted to measure procedural learning: an assembly learning task, requiring primarily motor skills, and a mirror drawing learning task, which requires greater cognitive functions. Procedural learning was defined in terms of the improvement in performance on each task over repeated trials, as reflected by higher scores and greater efficiency and speed.

#### The Assembly Learning Task

The assembly learning task used in this study is a motor procedural learning task adapted from the assembly subtest of the Purdue Pegboard Test [35, 36]. The Purdue Pegboard has been widely used as a predictor of the presence and laterality of cerebral lesions in adults [37, 38]. The validity and reliability of the Purdue Pegboard Test in population of 5–14 year-old children has been proved [39]. In addition, recent studies looking at motor development and motor impairment within samples constituted by children used the Purdue Pegboard Test as a measure [40, 41]. The test involves the assembly of small metal pins (pegs), collars and washers in groups of four onto a pegboard. The order of assembly is pin-washer-collar-washer. Children are asked to alternate the hand for each component added. Right-handed children are asked to start with the right hand and left-handed children, with the left. The score is the number of assembled pieces (NAP) in 60 seconds.

The adaptation made in this study was that participants were asked to repeat the task four times: three times consecutively, and the fourth after a 10-minute nonverbal interference task. This adaptation was made in order to evaluate the process of procedural learning. Three external researchers (not involved in this study) who are experts on the assessment of procedural learning agreed to evaluate the validity of the task for this purpose. It was found agreement between the three of them. This adaptation has been used as a measure of procedural learning in a recent study [42].

Percentage improvement in NAP scores were calculated as, for example: %NAP_1-2_ = [(NAP_2_ –NAP_1_) 100] / NAP_1_, where the subscripts 1 and 2 refer to the first and second trials, respectively. NAP_1_ reflects the baseline scores (basal skills); NAP_2_ and NAP_3_ reflect performances after repetition; NAP_4_ reflects performance after interference. Similarly, %NAP_1-2_, %NAP_1-3_, and %NAP_1-4_ measure improvement for the first to the second, third and fourth trials, respectively. In addition to NAP scores, we recorded whether children learned the assembly order and whether they alternated the hand for each component added. Motor skill improvement has been measured by percentage improvement in other studies [43].

#### The Mirror Drawing Learning Task

The mirror drawing learning task used in this study was based on the Mirror Drawing Test [44–47]. The original task involves drawing a five-pointed star through a mirror. The test has been used to study preserved skills in individuals with anterograde amnesia [12], in neuroimaging studies [48], in research into perceptual motor skills [49] and in research on anxiety and mental fatigue caused by prolonged cognitive load [50–52]. A computerized version of the test is included in *Physiology of Behavior* [53]. Balinsky & Stone [54] established the normal performance values for high-school children at mirror drawing a six-pointed star, and Clinton [47] published norms on mirror-drawing for children by age (7–13 years old) and sex.

In this study we used an adapted task with the following characteristics. Instead of a star, participants were asked to draw a line in a single stroke. The drawing sheet was under a small "table" where it was not directly visible but where it could be seen, together with the hand, by means of a mirror. Each child was tested individually, and only provided with verbal instructions when they had the set-up in front of them. Specifically, the task involved drawing a line in the space contained between two straight, parallel lines, if possible without lifting the pencil from the drawing sheet. In the case of right-handed children, the drawing stroke was to run downward from left to right. Left-handed children (8 participants) were asked to draw downward from right to left. Each subject performed the task four times: three trials in a row and the last one after a period with interference. Three external researchers with extensive experience using the Mirror Drawing Test [44–47] agreed on the validity of our adaptation for the measurement of procedural learning. This adaptation has been used as a measure of procedural learning in a recent study [42]. Even with its simplified and adapted form, the mirror drawing task used here is considerably more complex than the assembly learning task.

For each trial, we recorded the time required to draw the line (from start until the end of the parallel lines was reached) and the number of errors (leaving the parallel lines and/or skipping stretches). Number of errors has been proved to have an important influence on the motor skills acquisition of children [55]. Errors were manually counted independently by two of the authors and only in case of disagreement, the third author was involved. If a subject had required more than three minutes and 30 seconds to complete the trial, the trial was considered failed. From the time spent (T) and the number of errors (NE) we calculated an efficiency index (EI) as: EI = 10 (40—NE / T). A credit of 40 points was assigned for completion of the line, the precise value being chosen on the basis of the maximum number of errors made by any one subject (which was 38).

Improvement was measured as the percentage improvement [43] in time and also as the percentage improvement in efficiency index. Percentage time improvement (% T), for example between trials one and two, was calculated as: %T_1-2_ = [(T_1_ –T_2_) 100] / T_1._ The corresponding percentage efficiency improvement (%EI) was calculated as: %EI_1-2_ = [(EI_2_ –EI_1_) 100] / EI_1_.

As with the Assembly Learning Task, measurement and score subscripts refer to sequential order of trials; T_1_ and EI_1_ reflect basal skill; T_2_, EI_2,_ T_3_ and EI_3_ reflect skills developing with practice; and T_4_ and EI_4_ reflect performance after interference. Similarly, %T_1-2_ and %EI_1-2_, %T_1-3_ and %EI_1-3_, and%T_1-4_ and %EI_1-4_ reflect the improvement from the first to the second, third and fourth trials, respectively.

### Statistical analysis

The influence of repeated practice on performance was evaluated by means of analysis of variance (ANOVA) with repeated measures, using Bonferroni correction for Post hoc test, when the differences between pairs were normally distributed; and paired-sample tests (Wilcoxon signed-rank Test or Sign Test) were used with non-parametric data, for which the scores on different trials of the same test were considered to be paired samples.

In order to examine the relationship between age and performance, we calculated Pearson and Spearman correlation coefficients and analyzed differences with the Mann-Whitney U test (for two independent samples). In addition, a simple linear regression test (Theil's Incomplete Method) was used. Correlations between the scores of both tasks were evaluated by Spearman coefficient.

## Results

### Assembly Learning Task

Scores obtained by participants in their first trial were consistent with those in the Purdue Pegboard Examiner Manual for children between 6–12 years old [35]. All participants learned the order in which the pieces had to be assembled, and 90.1% of them properly alternated the hand for each component added. Number of assembled pieces (NAP) scores increased by 18.5% after three trials and 20.7% after the interference process.

**Fig 1:**
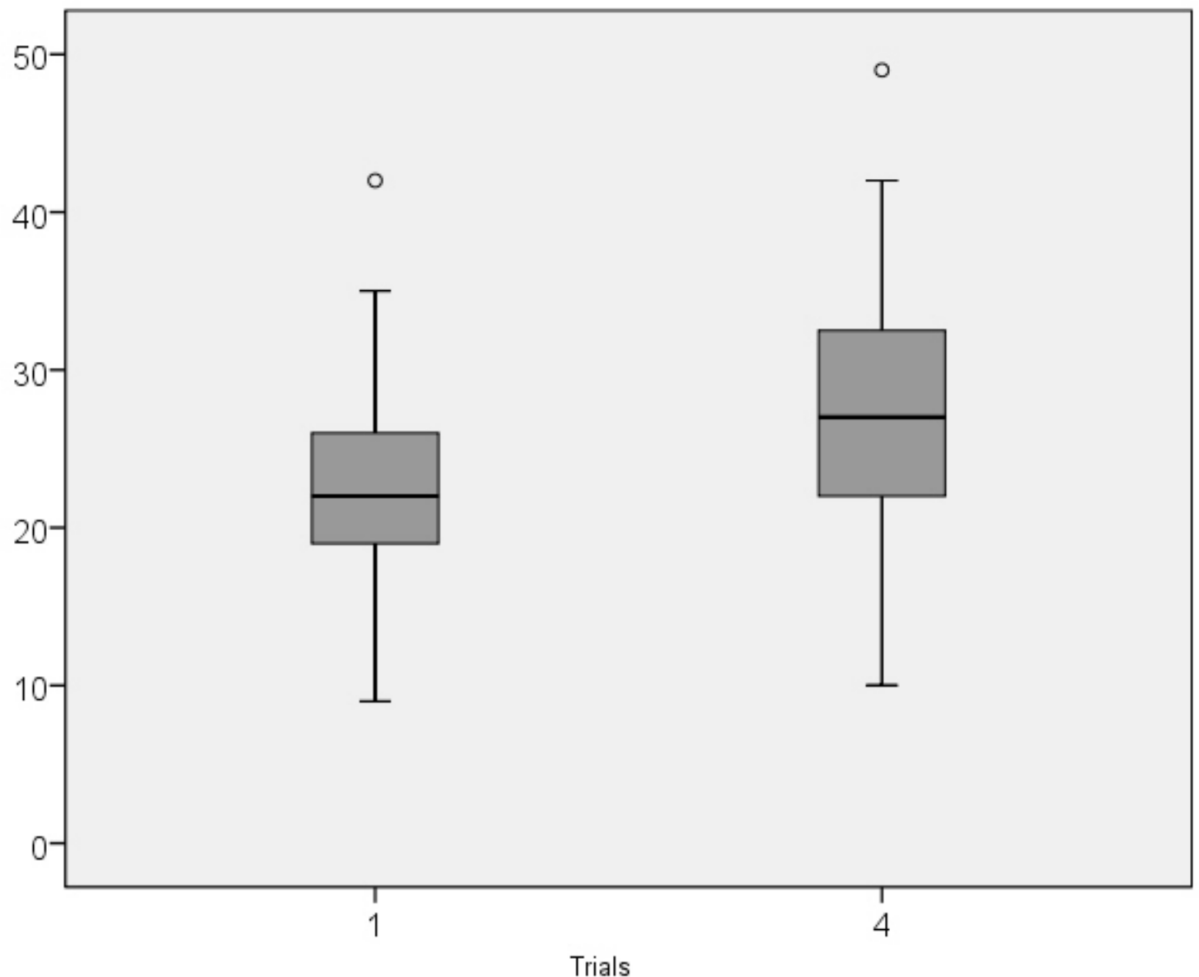
Performance on Assembly Learning Task. Median scores of Number of assembled pieces in trials 1 and 4. o, outliers.

As shown in Table 2 and Fig 1, typically developing children, as a group, achieved higher NAP scores through repetition. There are significant differences between NAP_1_ and NAP_2_ in all age ranges except the 10-year-old group; between NAP_1_ and NAP_3_ in all age groups; and between NAP_1_ and NAP_4_ in all age ranges except the 7-year-old group (see descriptive statistics in S1 Appendix).

**Table 2:** Performance on Assembly learning task and Mirror drawing learning task.

	Trial	Descriptive statistics [Mean (SD)]	Trial				
			1	2	3	Int.	4
**Assembly Learning Task**							
*Number of assembled pieces*	1	22.56 (5.61)	**-**	**	*	**-**	**
	2	25.08 (6.94)			**	**-**	**
	3	26.74 (7.41)				**-**	
	Int.					**-**	
	4	27.23 (7.77)				**-**	**-**
**Mirror Drawing Learning Task**							
*Time (seconds)*	1	82.35 (65.73)	**-**	**	**	**-**	**
	2	56.49 (53.07)			**	**-**	**
	3	33.54 (32.29)				**-**	**
	Int.					**-**	
	4	25.52 (22.21)				**-**	**-**
*Efficiency index*	1	5.72 (5.16)	**-**	**	*	**-**	**
	2	9.62 (8.26)			**	**-**	**
	3	17.52 (13.54)				**-**	**
	Int.					**-**	
	4	23.17 (15.52)				**-**	**-**

*Note*. Analysis of variance with repeated measures (parametric data) and evaluation of differences between pairs of trials (non-parametric). Int., period of interference.

** *p* < .01.

* *p* < .05.

**Fig 2:**
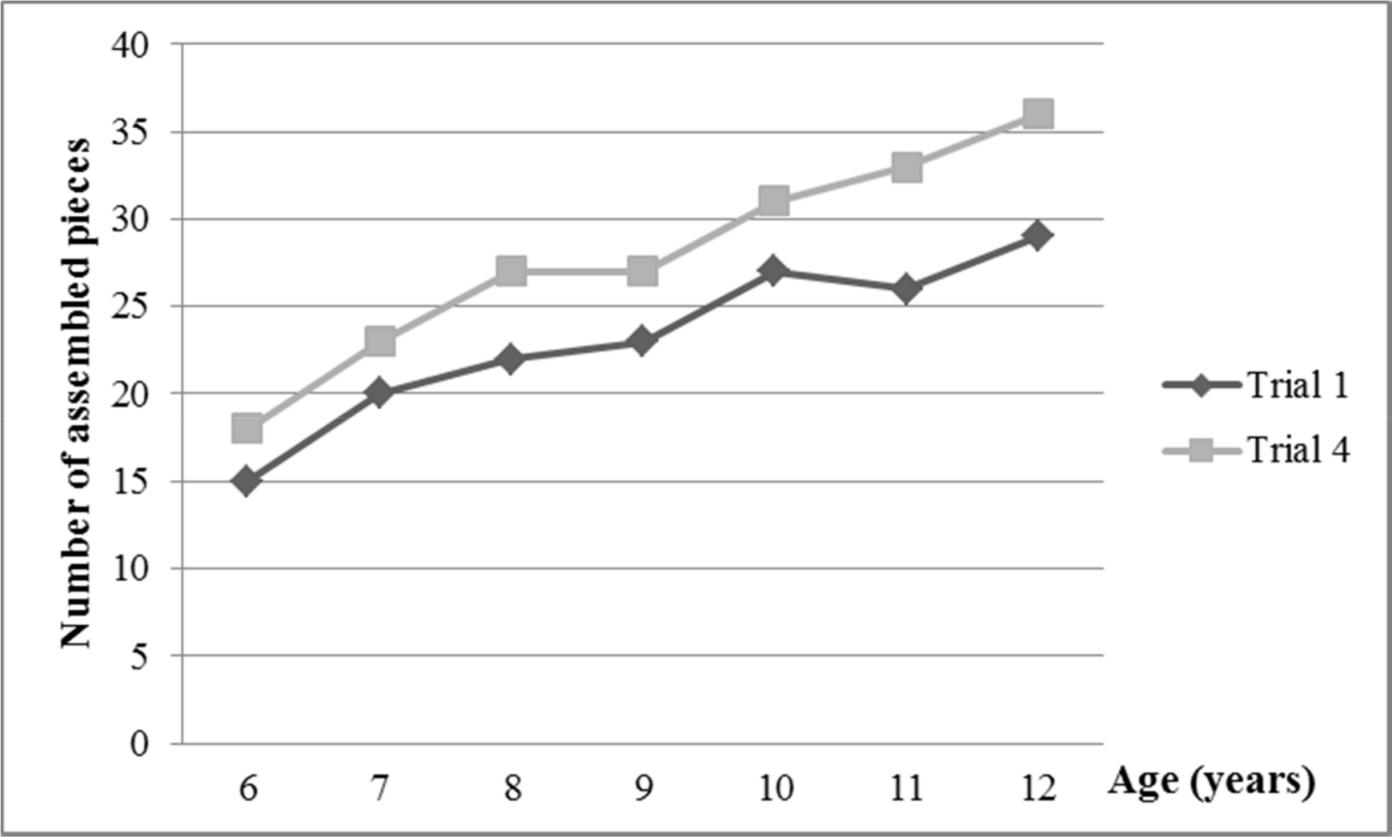
Performance on Assembly Learning Task by age. Legend: Number of assembled pieces (mean scores) in trials 1 and 4.

**Fig 3:**
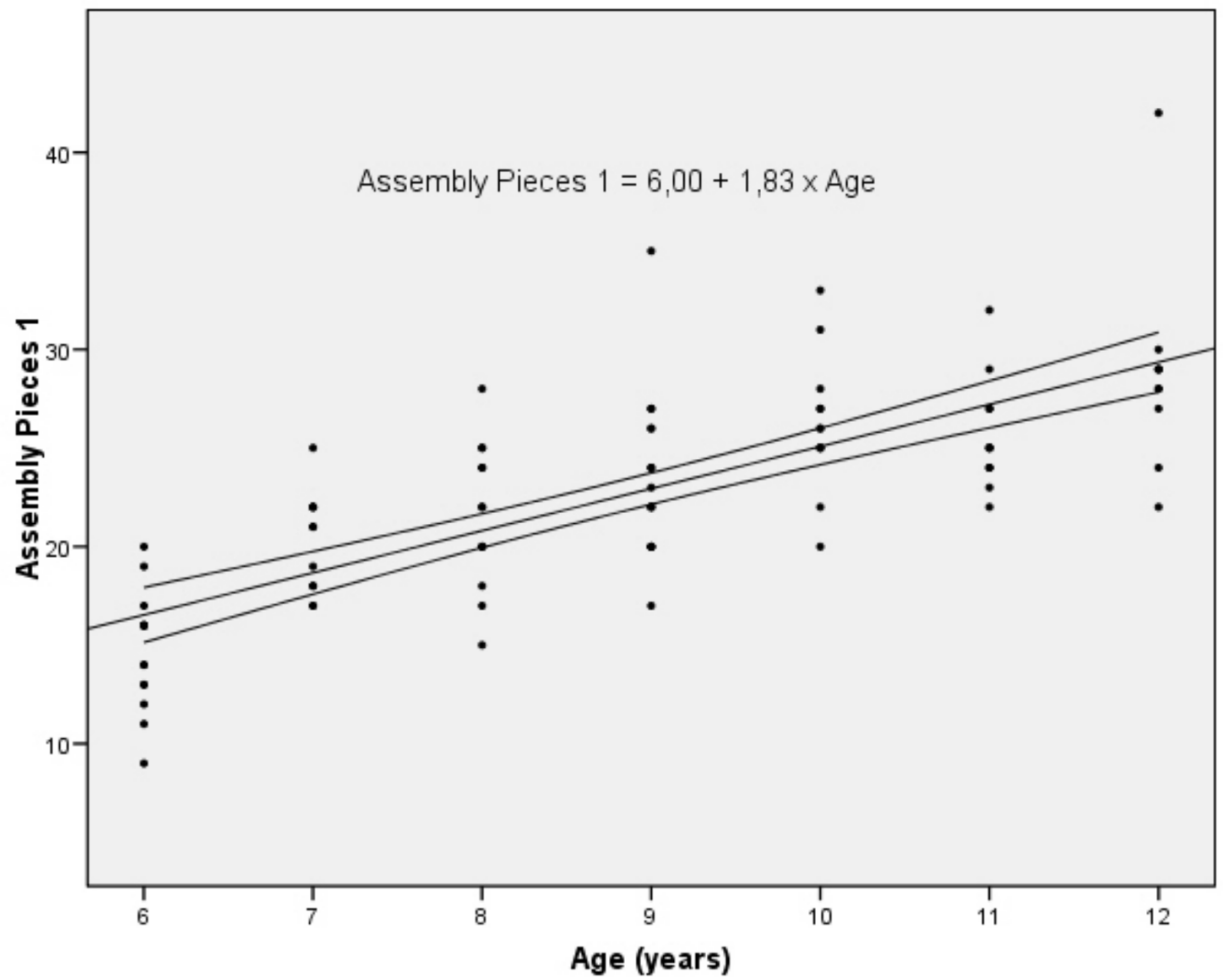
Number of assembled pieces in trial 1 by age. Legend: Regression line (with upper and lower confidence intervals).

Older children fitted more pieces than younger ones in all trials (Fig 2 and S1 Appendix). There was strong correlation (r_s_ > 0.7) between age and performance in each trial, and coefficients in regression studies were also significant (Fig 3 and Table 3).

**Table 3:** Relationship between performance and age.

Equation of the Regression line	R^2^
***Assembly Learning Task***	
NAP 1 = 6.00 + 1.83 × age (*p* < .001)	.40 *
NAP 2 = 7.00 + 2.00 × age (*p* < .001)	.36 *
NAP 3 = 7.00 + 2.17 × age (*p* < .001)	.38 *
NAP 4 = 14.00 + 1.5 × age (*p* < .001)	.30 *
***Mirror Drawing Learning Task***	
T 1 = 200.67–15.33 × age (*p* < .001)	.12 *
T 2 = 126.83–9.33 × age (*p* < .001)	.17 *
T 3 = 65.50–4.33 × age (*p* < .01)	.10 *
T 4 = 23.96–0.58 × age (*p* = .06)	.02 **
EI 1 = - 1.60 + 0.70 × age (*p* < .001)	.10 *
EI 2 = - 8.93 + 1.87 × age (*p* < .001)	.14 *
EI 3 = - 8.93 + 2.49 × age (*p* < .001)	.07 **
EI 4 = 0.24 + 2.28 × age (*p* < .05)	.04 **

*Note*. Equations of the regression lines. NAP, number of assembled pieces; T, time; EI, efficiency index; 1, first trial; 2, second trial; 3, third trial; 4, fourth trial; R^2^, coefficient of determination (Theil's Incomplete Method).

** *p* < .01.

* *p* < .05.

In order to simplify analysis of the relationship between improvement (improvement percentages) and age, participants were grouped into the three age ranges by which elementary school education is divided in Spain: 6–8, 9–10 and 11–12 years. Children in the 11–12 year-old group had higher NAP improvement percentages than children in the 6–8 and 9–10 year-old groups. Specifically, there were significant differences in %NAP_1-2_ between 6–8 and 11–12 year-olds; in %NAP_1-2_ between 9–10 and 11–12 year-olds; and in %NAP_1-3_ between 6–8 and 11–12 year-olds (Table 4). Thus the older group demonstrated larger improvement of skills than the younger groups. However, 9–10 year-olds did not have higher improvement percentages than 6–8 year-olds (Table 5).

**Table 4:** Evaluation of the differences between means.

	*Assembly Learning Task*			*Mirror Drawing Learning Task*			
	11–12 year-olds			11–12 year-olds			
	*% NAP*_*1-2*_	*% NAP*_*1-3*_	*% NAP*_*1-4*_		*% EI*_*1-2*_	*% EI*_*1-3*_	*% EI*_*1-4*_
**6–8 years**				**6–8 years**			
*% NAP*_*1-2*_	**			*% EI*_*1-2*_			
*% NAP*_*1-3*_				*% EI*_*1-3*_		**	
*% NAP*_*1-4*_				*% EI*_*1-4*_			**
**9–10 years**				**9–10 years**			
*% NAP*_*1-2*_	**			*% EI*_*1-2*_			
*% NAP*_*1-3*_		*		*% EI*_*1-3*_		**	
*% NAP*_*1-4*_				*% EI*_*1-4*_			

*Note*. Analysis of variance with repeated measures (parametric data) and evaluation of differences between pairs of trials (non-parametric); %NAP, Percentage of improvement in Number of assembled pieces; % EI, Percentage of improvement in Efficiency Index; subscript 1, first trial; subscript 2, second trial; subscript 3, third trial; subscript 4, fourth trial.

** *p* < .01.

* *p* < .05.

**Table 5:** Mean improvement percentages by age group.

	6–8 year-olds	9–10 year-olds	11–12 year-olds
***Assembly Learning Task***	**[Mean (SD)]**	**[Mean (SD)]**	**[Mean (SD)]**
*% NAP*_*1-2*_	11.77 (13.81)	10.76 (16.62)	14.75 (11.18)
*% NAP*_*1-3*_	18.67 (15.52)	16.00 (16.79)	23.47 (13.85)
*% NAP*_*1-4*_	21.74 (20.21)	16.98 (16.70)	24.56 (13.95)
***Mirror Drawing Learning Task***			
*% EI*_*1-2*_	201.97 (230.29)	74.47 (91.01)	81.21 (68.38)
*% EI*_*1-3*_	484.86 (388.68)	234.12 (197.24)	71.82 (28.93)
*% EI*_*1-4*_	741.90 (515.93)	390.23 (420.55)	100.42 (92.77)

*Note*. % NAP, Percentage of improvement in Number of Assembled pieces; % EI, Percentage of improvement in Efficiency Index.

### Mirror Drawing Learning Task

The difficulty of the mirror drawing learning task was reflected by the fact that 16% of the participants required more than three minutes and 30 seconds to complete the task the first time they faced it; after four trials this percentage decreased to 8%.

**Fig 4:**
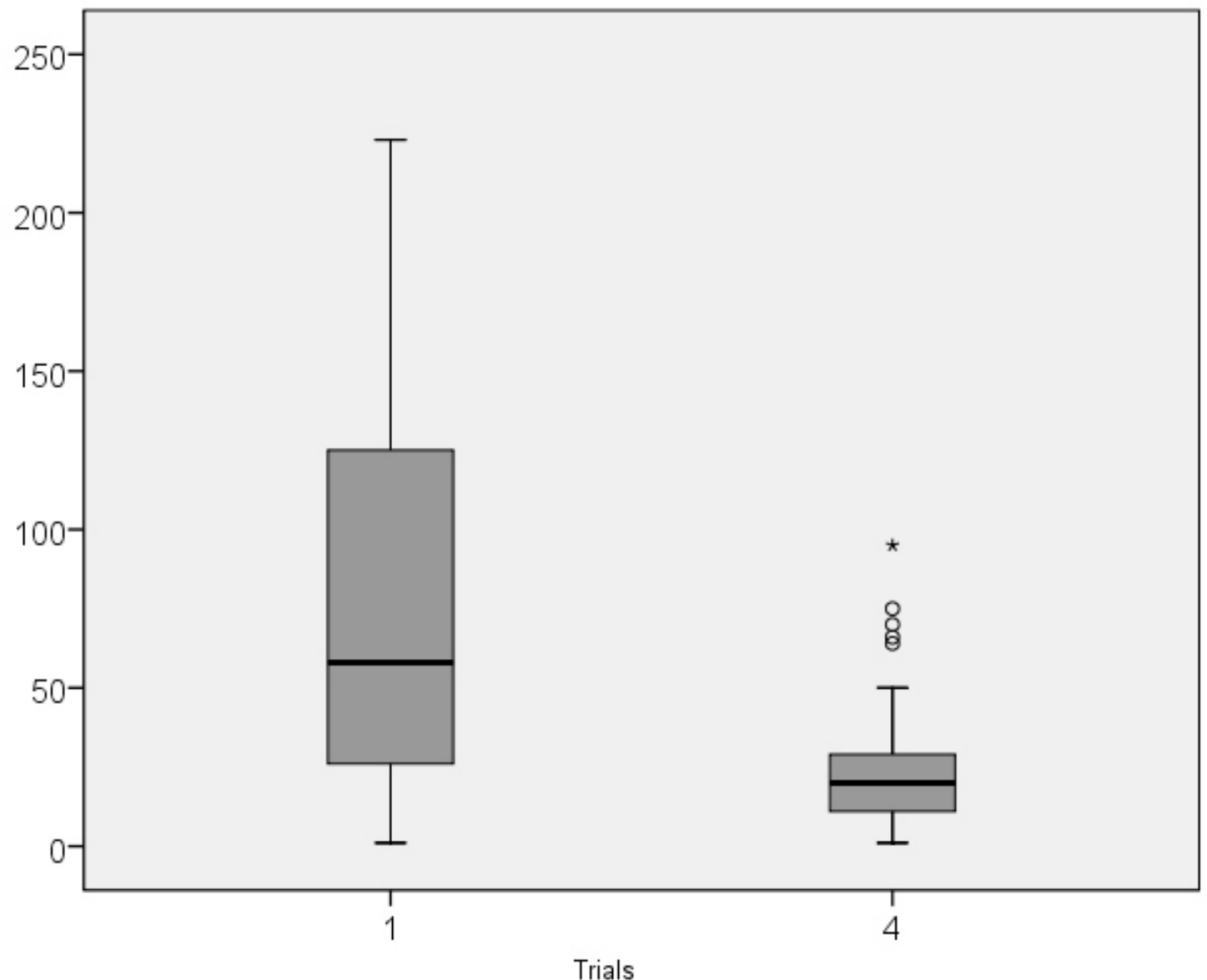
Median scores in Mirror Drawing Learing Task completion times in trials 1 and 4. Legend: o, outliers; *, extreme outliers.

**Fig 5:**
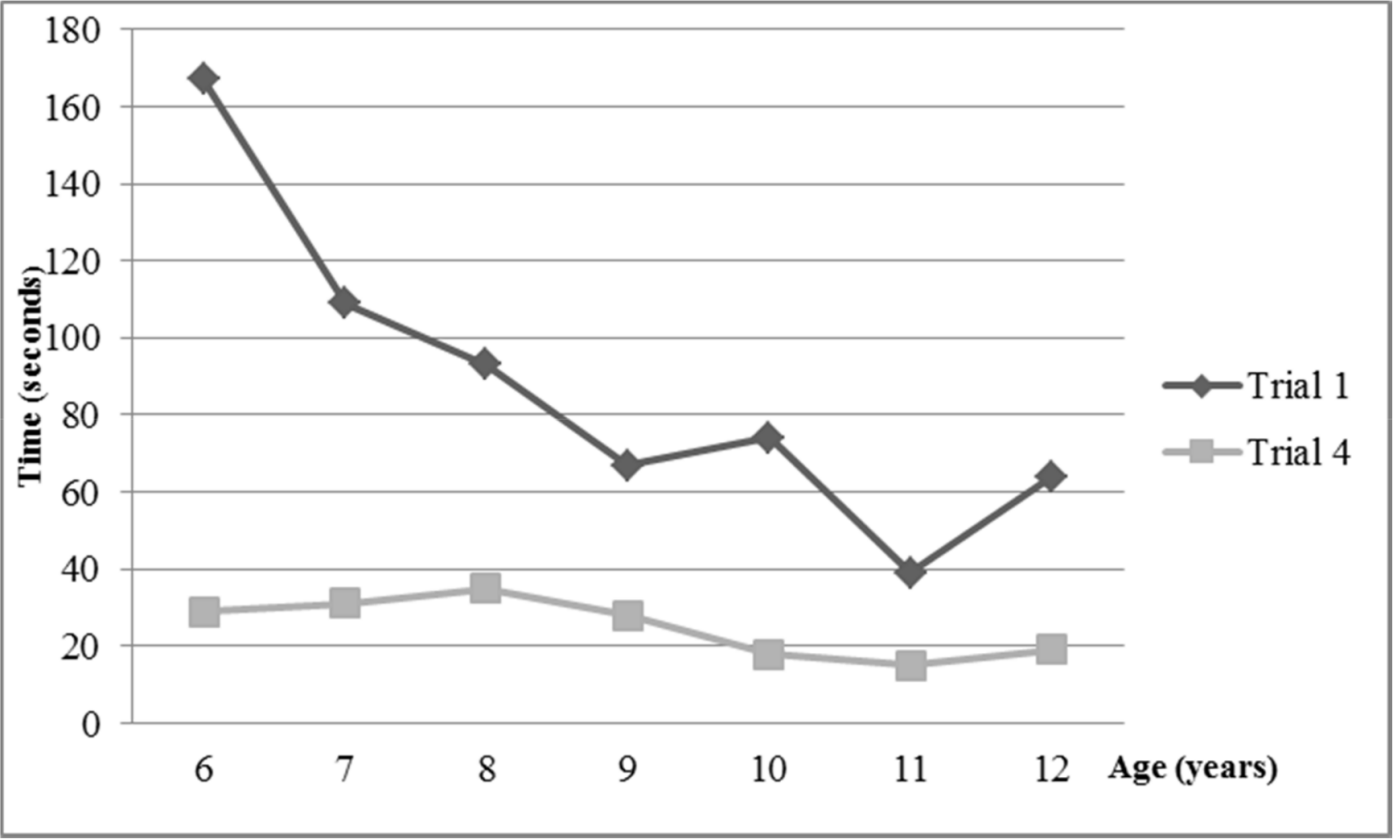
Mirror drawing learning task completion times in trials 1 and 4 by age (mean times).

Completion time decreased over the course of the first three consecutive trials as well as in the final post-interference trial (Table 2 and Fig 4). There were significant differences between T_1_ and T_4_ in all age ranges (see descriptive statistics in S1 Appendix). Shorter completion times correlated with older age (Table 3 and Fig 5): the correlations between age and T_1_ and T_3_ were moderate, and correlation between age and T_4_ was strong.

**Fig 6:**
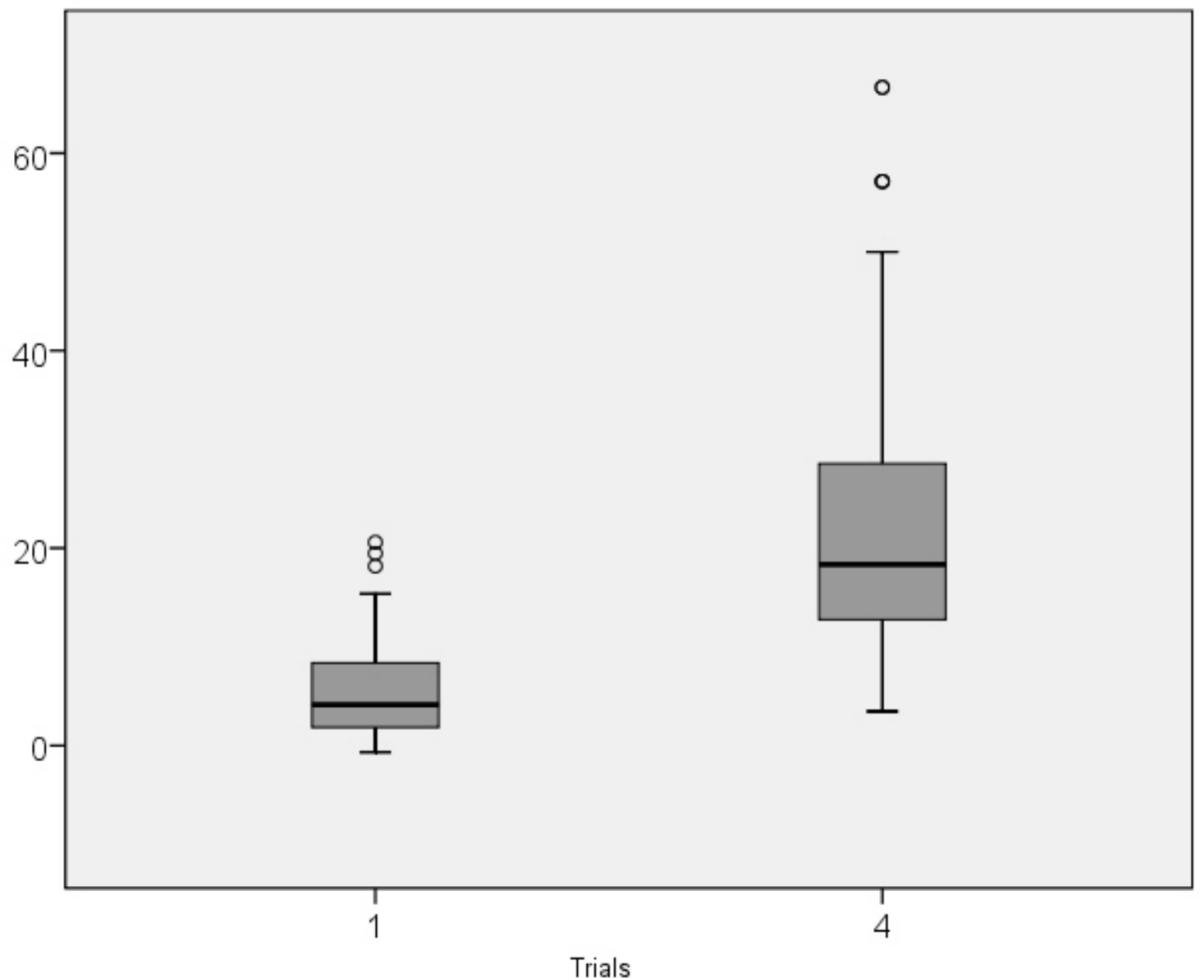
Mirror drawing learning task efficiency indices in trials 1 and 4. Legend: o, outliers.

**Fig 7:**
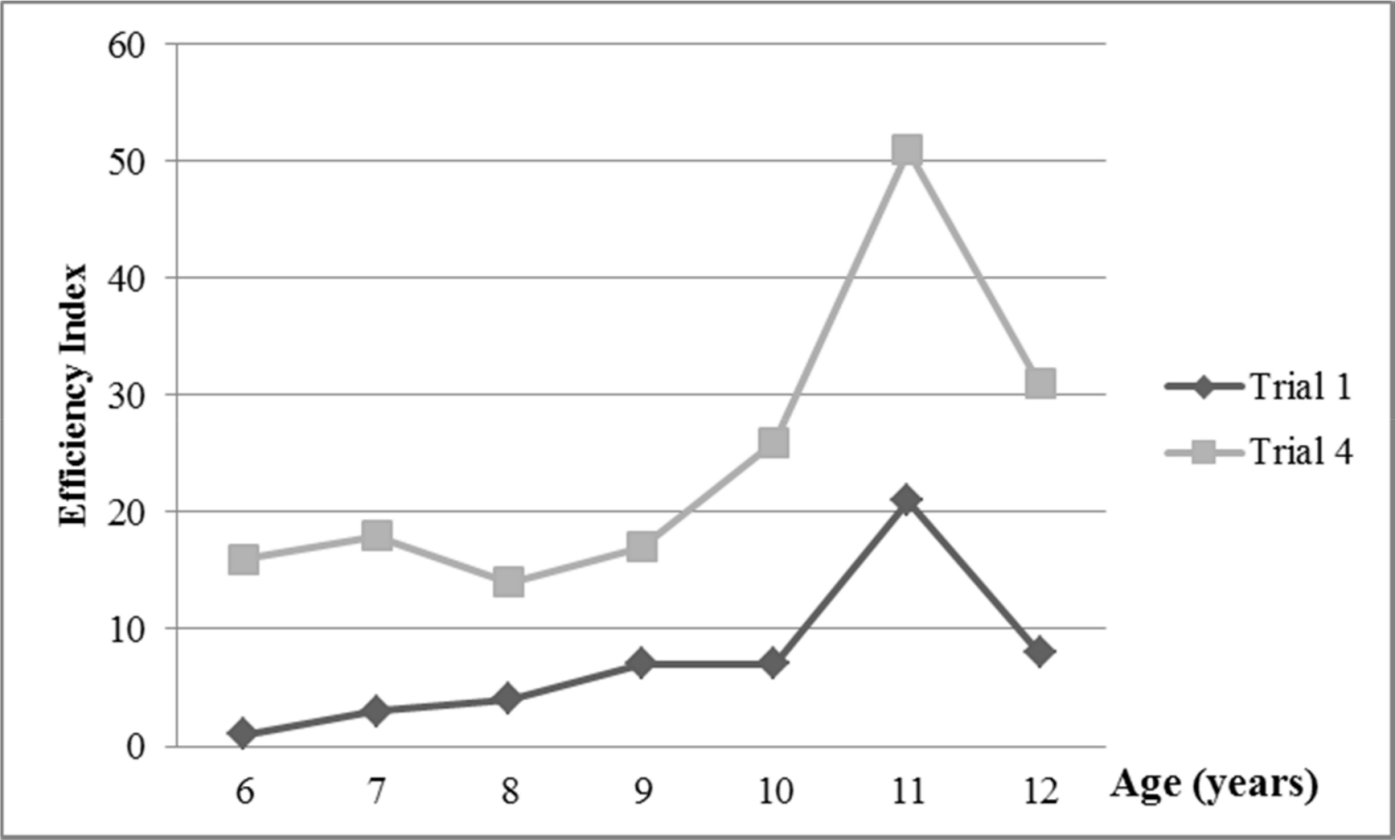
Mirror drawing learning task efficiency indices by age (mean values).

With repetition, the typically developing children in our sample became more efficient (measured by Efficiency Index-EI) at the mirror drawing learning task (Table 2 and Fig 6). There were significant differences between EI_1_ and EI_3_ and between EI_1_ and EI_4_ in all age groups (see descriptive statistics in S1 Appendix). There was a strong correlation between age and EI_1_ and EI_2_ and moderate correlation between age and EI_3_ and EI_4_; the increase in EI with age is shown in Fig 7. Note that EI increases specially at the stage of 11 years; an explanation for this result could be that at this stage the proportion of males is larger than the number of females. Equations of the regression lines are shown in Table 3.

Efficiency improvement percentages decreased with age (Table 5): %EI_1-3_ was higher in 6–8 year-olds than in 11–12 year-olds (*p* < .01); %EI_1-3_ was higher in 9–10 year-olds relative to 11–12 year-olds (*p* < .01); and %EI_1-4_ was higher in 6–8 year-olds than 11–12 year-olds (*p* < .01) (Table 4).

### Relationship between scores in the assembly learning task and the mirror drawing learning task

Correlations between the scores in the assembly learning task and the mirror drawing learning tasks were found in the 6–8 year-old group and in the 11–12 year-old group (S2 Appendix).

## Discussion

### Influence of training on procedural learning

In our study, three consecutive trials at a procedural task increased speed, score and efficiency. Improvement by repetition occurred in both of the tasks evaluated: assembly, which requires motor skills, and mirror drawing, which requires more cognitive functions. Although it was possible to identify differences between groups at a criterion of significance of *p* < .01, it should be noted that standard deviations in performance measurements in this study tended to be high, indicating the need for a large sample size in any future studies along these lines.

In the assembly learning task, participants were allowed a fixed 60-second time period in which to do the assembly trial. Determination of how much time to allow was not part of the experimental design, but 60 seconds is, perhaps, rather short to permit maximal improvement between the consecutive trials. Nevertheless, statistically significant performance differences were observed. In the second task used, the mirror drawing learning task, repetition reduced both the time required and, to a greater degree, the number of errors.

A ten-minute period of interference with procedural memory tasks, which was imposed after the three consecutive trials, resulted in only a slight stalling in the rate of improvement in performance. Note that this interference period did not impair performance in the tasks, suggesting that repeating a trial three times is sufficient to significantly improve it.

Our results are consistent with published reports. In a longitudinal study of implicit memory, as a secondary finding it was noted that training on a computerized serial reaction-time task reduced the reaction time [56]. Ward et al. [15], using a rotary pursuit task and a mirror reading test, studied procedural memory in children with amnesia. Participants repeated the tasks four times in a row, and a fifth time after a 30 minute break. As an incidental finding, the authors report that for both tasks the performance of the typically developing children evaluated as controls improved through training. Learning was defined as a decrease in the time required and a decrease in number of errors; the authors do not specify whether one of these measures predominated. Interestingly, children with amnesia due to traumatic brain injury had preserved procedural memory skills.

Prehn-Kristensen et al. [57] used a learning session comprising ten trials at a mirror tracing task as part of a study into the influence of sleep on emotional declarative and procedural memory. The authors noted that participants got faster and made fewer errors as the learning session progressed. Sullivan et al. [58] examined the practice of 200 trials of a discrete arm movement with specific spatiotemporal parameters, finding that all participants (twenty adults and twenty 8–14 year-old children), regardless the beedback given, improved accuracy and consistency across practice trials. In a similar design, Murphy et al. [59] analyzed the effects of three types of augmented feedback on the acquisition and retention of a stationary kicking skill through repetition; all children (third grade elementary students) showed significant performance improvement independently of the type of feedback. The effect of part and whole practice in motor learning was recently studied in a sample of first, third and fifth grade children; the results suggested that all students improved their juggling skills through repetition (both in part and whole practice conditions) [60]. The effects of practice on the acquisition of a simple grapho-motor task has been measured in a sample of 5–8 year-old children [61]. Vicari et al. [62, 63] investigated the effects of specific types of tasks on the efficiency of implicit procedural learning in the presence of developmental dyslexia. Participants and matched normal-reader controls were given the serial reaction-time test and the mirror-drawing test, in which implicit knowledge was gradually acquired across multiple trials. In the control participants, the speed of tracing increased and the number of errors decreased with repetition. Elbert et al. [64] reported that, with procedural tasks, the number of errors was initially high but could decrease with repetition to the standards of an expert. Similar results have been reported in studies of motor learning in adults. Reddon et al. [65] evaluated temporal stability and efficacy of lateralization for the Purdue Pegboard test in 26 adult participants assessed five-times at weekly intervals; the performance of female participants on the assemblies subtest was found to increase significantly.

### Influence of age on the development of procedural memory

Procedural memory involves the learning of a cognitive process (which is then done with greater efficiency) or of a motor procedure (which can then be done with greater spatial and temporal accuracy). When a cognitive or motor exercise is repeated sufficiently to automate it, the attention required to carry it out decreases to the point that it no longer requires awareness to be carried out [10].

In the present study, we found that age affected basal performance in motor procedures (assembly) and affected the basal time requirement and basal efficiency in the use of cognitive strategies (mirror drawing). These results are consistent with those reported by other researchers. Several studies conclude that the basal ganglia, which are crucial at the neural level for implicit learning, attain highest development between 7.5 and 10 years of age [66, 67].

Nelson [68] assessed visuomotor sequence learning in 4- to 10-year-old children using a serial reaction-time (SRT) task to which half of the participants had previous exposure. Age-related differences were found in explicit knowledge but also, to a lesser degree, in implicit knowledge. Differences on the SRT task performance across age groups have been reported by other authors (Du, 2012). Thomas et al. [69] studied performance at an implicit learning task (requiring a bimanual response) by 7-year-old children and adults aged between 23 and 33 years. Various age-related differences were found. Similarly, De Guise & Lassonde [70] found a directly proportional relationship between age and implicit learning of a two-hand (but not single-hand) response. Cognitive procedural learning among children and adolescents (9–18 years old) with or without spastic cerebral palsy was analyzed: an age effect was found among typically developing children on Probabilistic Classification Learning (PCL) [71].

In contrast, Meulemans and Van der Linden [72] found no age-related differences in SRT performance in a sample of 6- and 10-year-old children and adults. Karatekin, Marcus, & White [73], also using SRT tests, found little evidence of differences in incidental learning capacities (sequence-learning indices) during middle childhood and adolescence. Their results suggested that the ability to learn a sequence rapidly under incidental conditions is mature by 8 to 10 years of age. In a recent study, examining motor and cognitive procedural learning (SRT and PCL) the results showed similar learning effects in three age groups (8, 10 and 12 years old) [74]. Schacter & Moscovitch [75] have suggested that the brain structures involved in implicit memory develop before those crucial for explicit memory.

That age influences baseline procedural skills as a consequence of neurobiological development and experience would not be surprising [26], although there is not an over-abundance of studies that specifically analyze and demonstrate it. However, there is even less research on how age affects an individual's capacity to improve, by means of repetition, his or her performance of procedural tasks. In the present study, we calculated percentage improvement from the first to successive trials and compared these percentages for our participants divided into three age groups (6–8, 9–10 and 11–12 year-olds). At the assembly learning task, 11–12 year-olds improved faster than 6–8 year-olds. At the predominantly cognitive task of mirror-drawing, however, 6–8 year-olds improved in efficiency faster than the 11–12 year-olds. This latter result may be a consequence of a ceiling effect in the learning of mirror-drawing; ceiling effects have been reported for healthy subjects in various procedural memory tests, such as, the Porteus Maze [14, 76], the Pursuit Rotor test [14] and performing faster finger responses [77]. Other explanations are that implicit sequence learning depends on the structure of response execution and on the relative task difficulty [78, 79], and that the greatest learning is exhibited at an optimal level of cognitive challenge [80]. In view of the importance of age in the development and maturation of the capacity to learn cognitive and motor skills, it would be interesting to evaluate the learning of assembly and mirror-drawing tasks in children under the age of six years.

Although it can be difficult to verbalize the steps followed in an efficient performance of a procedure or to justify one order rather than another [81], this does not necessarily mean, that it is not possible to become aware of how to perform a strategy. Making the sequence of actions that constitute a task explicit, for example, by prior or *in situ* verbalization, can help children with difficulties in the acquisition and automation of procedures [82].

A limitation of this study is that, the small sample size of a wide age range, resulted to a few children in each age group of the study.

## Conclusions

These findings indicate that in the population of 6–12 years old typically developing children, three consecutive trials at a procedural task increased speed and efficiency. And that age affected basal performance in motor-cognitive procedures.

## Supporting Information

S1 Appendix Mean and standard deviation of the measures of performance by age range. (DOCX)

S2 Appendix Correlation between Assembly Learning Task scores and Mirror Drawing Learning Task scores. (DOCX)
