# Supplementary material for: Acquisition of Motor and Cognitive Skills through Repetition in Typically Developing Children
Source: PLoS One. 2016 Jul 6;11(7):e0158684. doi: 10.1371/journal.pone.0158684 (PMC4934913; doi:10.1371/journal.pone.0158684)
Supplement: S1 Appendix — (DOCX) [file pone.0158684.s001.docx]

|  | Age (years;months) | | | | | | | |
| --- | --- | --- | --- | --- | --- | --- | --- | --- |
|  | 6;0-6;11  (n=17) | | 7;0-7;11  (n=11) | 8;0-8;11  (n=13) | 9;0-9;11  (n=16) | 10;0-10;11  (n=12) | 11;0-11;11  (n=11) | 12;0-12;11  (n=10) |
| *Assembly Learning Task* |  |  |  |  |  |  |  |  |
| Number of Assembled pieces |  |  |  |  |  |  |  |  |
| Trial 1 | M  SD | 14.80  2.93 | 20.18  2.56 | 21.54  3.67 | 23.44  4.23 | 26.25  3.49 | 25.73  2.87 | 28.80  5.27 |
| Trial 2 | M  SD | 16.53  3.83 | 22.55  3.30 | 23.77  4.23 | 24.50  4.13 | 28.67  5.52 | 30.18  3.76 | 33.40  8.14 |
| Trial 3 | M  SD | 16.87  3.76 | 24.45  3.11 | 25.23  5.62 | 26.19  3.66 | 30.67  5.21 | 33.00  6.34 | 35.30  5.77 |
| Trial 4 | M  SD | 17.93  4.23 | 22.73  4.76 | 27.31  7.03 | 27.13  5.83 | 30.75  5.01 | 32.91  5.17 | 35.70  6.53 |
|  |  |  |  |  |  |  |  |  |
| *Mirror Drawing Learning Task* |  |  |  |  |  |  |  |  |
| Time |  |  |  |  |  |  |  |  |
| Trial 1 | M  SD | 167.00 44.39 | 108.57  57.89 | 92.75  72.23 | 67.29  50.08 | 73.50  58.30 | 38.73  47.58 | 63.50  66.21 |
| Trial 2 | M  SD | 110.75 43.15 | 86.44  75.35 | 70.25  59.63 | 57.14  57.20 | 38.00  17.95 | 22.73  23.15 | 28.00  19.81 |
| Trial 3 | M  SD | 54.67 62.00 | 41.80  19.52 | 43.46  30.17 | 35.73  38.57 | 24.58  10.83 | 17.55  14.88 | 18.40  9.58 |
| Trial 4 | M  SD | 28.56 17.39 | 31.36  37.32 | 34.62  24.30 | 28.33  21.98 | 18.08  8.57 | 14.89  19.00 | 18.80  9.44 |
| Efficiency Index |  |  |  |  |  |  |  |  |
| Trial 1 | M  SD | 1.26  1.06 | 3.16  2.94 | 3.95  3.31 | 6.92  4.49 | 7.15  5.80 | 20.89  17.71 | 8.21  7.51 |
| Trial 2 | M  SD | 2.22  1.96 | 4.74  4.27 | 3.95  3.53 | 10.82  7.89 | 12.25  8.00 | 28.09  21.05 | 17.76  13.97 |
| Trial 3 | M  SD | 12.09  7.05 | 9.02  4.77 | 9.60  6.51 | 23.65  19.53 | 19.17  11.67 | 36.84  26.70 | 28.80  21.87 |
| Trial 4 | M  SD | 16.43  8.07 | 18.54  8.05 | 14.14  11.09 | 16.70  9.23 | 26.28  11.39 | 50.71  28.94 | 30.66  27.07 |
