# Supplementary material for: Acquisition of Motor and Cognitive Skills through Repetition in Typically Developing Children
Source: PLoS One. 2016 Jul 6;11(7):e0158684. doi: 10.1371/journal.pone.0158684 (PMC4934913; doi:10.1371/journal.pone.0158684)
Supplement: S2 Appendix — (DOCX) [file pone.0158684.s002.docx]

|  | | Mirror Drawing Learning Task | | | | | | | | |
| --- | --- | --- | --- | --- | --- | --- | --- | --- | --- | --- |
|  |  | T_1_ | T_2_ | T_3_ | T_4_ |  | EI_1_ | EI_2_ | EI_3_ | EI_4_ |
| Assembly Learning Task | |  |  |  |  |  |  |  |  |  |
| 6–8 years | NAP_1_ | 0.61 ** | 0.53 ** |  |  |  | 0.60 ** | 0.53 ** |  |  |
|  | NAP_2_ | 0.46 * | 0.55 ** |  |  |  | 0.49 ** | 0.55 ** |  |  |
|  | NAP_3_ | 0.50 ** | 0.37 * |  |  |  | 0.46 * | 0.38 * |  |  |
|  | NAP_4_ | 0.49 ** | 0.48 ** |  |  |  | 0.53 ** | 0.44 * |  |  |
| 9-10 years | NAP_1_ |  |  |  |  |  |  |  |  | 0.39 * |
|  | NAP_2_  NAP_3_ |  |  |  |  |  |  |  |  |  |
|  |  |  |  |  |  |  |  |  |  |  |
|  | NAP_4_ |  |  |  |  |  |  |  |  |  |
| 11-12 years | NAP_1_ |  |  |  |  |  |  |  |  |  |
|  | NAP_2_ | 0.56 ** | 0.55 * | 0.58 ** | 0.46 * |  | 0.55 ** | 0.54 * | 0.57 ** |  |
|  | NAP_3_ | 0.48 * | 0.50 * | 0.48 * | 0.37 |  | 0.48 * | 0.50 * | 0.46 * |  |
|  | NAP_4_ |  | 0.47 * | 0.48 * | 0.46 * |  |  | 0.47 * | 0.47 * |  |

*Note*. NAP, Number of Assembled Pieces; T, time taken to complete the task; EI, Efficiency Index; subscript 1, first trial; subscript 2, second trial; subscript 3, third trial; subscript 4, fourth trial.

** *p* < .01

* *p* < .05
